# Real-World Associations Between Physical Activity, LDL Cholesterol, and Functional Performance in Primary Care: A Cross-Sectional Study

**DOI:** 10.3390/healthcare14111522

**Published:** 2026-05-30

**Authors:** Peter Marián Kalanin, Ivan Uher

**Affiliations:** 1 Department of General Medicine, Faculty of Medicine and MED–KAL, s.r.o., Pavol Jozef Šafárik University, Rastislavova 45, 041 80 Košice, Slovakia; peter.kalanin@upjs.sk; 2 Institute of Physical Education and Sport, Pavol Jozef Šafárik University, 041 80 Košice, Slovakia

**Keywords:** physical activity, primary care, LDL cholesterol, cardiovascular risk, functional performance, behavioral regulation, real-world data

## Abstract

**Highlights:**

**What are the main findings?**

- Higher self-reported physical activity (PA) levels were associated with lower low-density lipoprotein cholesterol (LDL-C) concentrations and better functional performance assessed using the Timed Up and Go (TUG) test.
- A graded cross-sectional association was observed, with progressively lower LDL-C levels and better TUG performance across increasing physical activity categories.

**What are the implications of the main findings?**

- PA assessment may provide clinically relevant information for cardiovascular risk and functional health evaluation in routine primary care.
- Regulatory processes are discussed only from a theoretical and hypothesis-generating perspective.

**Abstract:**

**Background**: Physical activity (PA) is associated with cardiometabolic health and functional performance, but evidence from real-world primary care populations simultaneously examining PA, low-density lipoprotein cholesterol (LDL-C), and functional performance remains limited. **Objective**: This study evaluated associations between PA, LDL-C, and functional performance in a real-world primary care cohort. **Methods**: This cross-sectional observational study included 863 adult primary care patients evaluated between February 2021 and March 2026. The overall cohort had a mean age of 52.4 ± 14.8 years, and 53.3% of participants were female. PA was assessed using self-reported activity categories (low, moderate, or high) obtained during a routine clinical evaluation. LDL-C concentrations were analyzed in the full cohort, while Timed Up and Go (TUG) functional performance assessment was available in an exploratory subgroup (*n* = 214). Multivariable regression analyses were adjusted for age, sex, body mass index (BMI), diabetes mellitus, and arterial hypertension in the TUG analysis. **Results:** Higher PA categories were associated with lower LDL-C concentrations across groups (*p* < 0.001). Mean LDL-C concentrations were 3.68 ± 1.05 mmol/L in the low PA group, 3.39 ± 0.97 mmol/L in the moderate PA group, and 3.12 ± 0.89 mmol/L in the high PA group. In the exploratory TUG subgroup, higher PA categories were also associated with better functional performance (*p* < 0.001). These associations remained significant after multivariable adjustment. **Conclusions**: Higher self-reported PA levels were associated with lower LDL-C concentrations in the full cohort and with better functional performance in the subgroup with available TUG data. Because of the cross-sectional observational design, these findings should be interpreted as associations and do not establish causality or directionality. Residual confounding, particularly from unavailable data on lipid-lowering medication use, smoking status, diet, socioeconomic status, and other cardiovascular risk factors, cannot be excluded. Future longitudinal studies using objective PA assessment, a more complete confounder assessment, and direct measurement of regulatory processes are warranted.

## 1. Introduction

Physical activity (PA) is a key determinant of cardiometabolic health and remains a central component of prevention and treatment strategies across medical disciplines. Despite advances in pharmacological management, cardiovascular diseases continue to represent the leading cause of global morbidity and mortality, with dyslipidemia—particularly elevated low-density lipoprotein cholesterol (LDL-C)—identified as a major modifiable risk factor [1,2,3]. Recent global analyses indicate that insufficient PA is associated with a substantial burden of cardiovascular and metabolic diseases [4,5]. A robust body of evidence shows that regular PA is associated with more favorable lipid profiles, including lower LDL cholesterol concentrations, as well as better functional capacity and physical performance [6,7,8]. These associations are biologically plausible and have been discussed in relation to mechanisms involving lipoprotein metabolism, insulin sensitivity, vascular function, and autonomic regulation [9,10,11]. In addition to metabolic health, PA is consistently associated with mobility, balance, and functional independence, particularly in aging populations [12,13]. However, despite strong evidence supporting the health relevance of PA, its long-term maintenance in real-world primary care settings remains inconsistent. A substantial gap persists between recommended PA levels and actual patient behavior [14,15,16]. Many individuals do not sustain regular PA over time despite awareness of its health relevance, suggesting that factors beyond knowledge and motivation may influence long-term PA behavior. Primary care provides a useful real-world context for examining associations between PA, cardiometabolic risk markers, and functional outcomes under routine clinical conditions. However, evidence from routine primary care populations examining PA in relation to both LDL-C and functional performance remains limited. This gap is clinically relevant because primary care settings routinely capture cardiometabolic risk and functional status, yet these outcomes are often examined separately rather than together. Therefore, the primary aim of the present study was to evaluate cross-sectional associations between PA, LDL-C concentrations, and functional performance in a real-world primary care population.

Importantly, the present study does not aim to establish causality or directionality. Rather, it evaluates cross-sectional associations between PA, LDL-C concentrations, and functional performance in a real-world primary care population. A secondary objective was to briefly discuss regulatory efficiency as a theoretical and hypothesis-generating construct for future research on sustained PA behavior. Regulatory efficiency was not directly measured in this study, and no conclusions regarding mechanisms can be drawn from the present data.

## 2. Materials and Methods

### 2.1. Study Design and Setting

This study was designed as a cross-sectional observational analysis using routinely collected clinical data from primary care. The real-world primary care setting reflects routine clinical practice and allows for evaluation of associations under standard clinical conditions. Because of the observational and cross-sectional design, the study was not intended to determine causal effects of PA on LDL-C concentrations or functional performance. The study followed the Strengthening the Reporting of Observational Studies in Epidemiology (STROBE) recommendations for observational research reporting. The completed STROBE checklist, including page and line references, is provided as Supplementary Table S1.

### 2.2. Participants

Adult patients (≥18 years) with available LDL-C measurements were eligible for inclusion in the analysis. Patients were consecutively included from the primary care database during routine clinical visits. No pre-selection was performed based on PA level, LDL-C status, cardiovascular risk profile, or functional performance. Inclusion criteria were: age ≥ 18 years, available PA classification, available LDL-C measurement, and available core demographic and clinical data required for the main analyses. For the functional-performance analysis, available Timed Up and Go (TUG) test data were additionally required. Exclusion criteria were: age < 18 years, missing PA classification, missing LDL-C data, missing key demographic or clinical variables required for the main analyses, and inability to classify the patient into one of the predefined PA groups. No formal a priori sample-size calculation was performed because this was a retrospective cross-sectional analysis of an available real-world primary care cohort. The final sample size was determined by the number of eligible adult patients with available PA classification, LDL-C measurement, and required clinical data during the study period. Therefore, the sample size reflects the available eligible cohort rather than a prospectively estimated target sample. The available cohort size was considered adequate for multivariable observational analyses given the number of included predictors and the overall sample distribution.

### 2.3. Data Collection

Data were obtained from routine clinical records in a primary care environment. All variables were collected as part of a routine clinical assessment and were anonymized prior to analysis. Clinical data were extracted from electronic medical records by trained personnel and included routinely documented variables recorded during patient visits. Demographic and clinical variables included age, sex, body mass index (BMI), presence of hypertension, and diabetes mellitus status. Hypertension was defined as the presence of a documented clinical diagnosis of hypertension and/or current antihypertensive treatment recorded in the medical record. Diabetes mellitus was defined as the presence of a documented clinical diagnosis of diabetes mellitus and/or current glucose-lowering treatment recorded in the medical record. Lipid-lowering medication use, smoking status, socioeconomic status, dietary variables, alcohol use, cardiovascular disease history, thyroid disease, and renal disease were not systematically available in the routine primary care dataset and therefore could not be included in the baseline table or multivariable adjustment models. LDL-C values were obtained from standard biochemical analyses performed in certified laboratories. LDL-C was measured using an enzymatic colorimetric assay on a cobas c clinical chemistry analyzer with LDL-Cholesterol Gen.3 reagents (Reche Diagnostics GmbH, Mannheim, Germany).

### 2.4. Physical Activity Assessment

Physical activity was assessed using patient self-reports as part of a routine clinical evaluation. Patients reported their average weekly duration of moderate-to-vigorous PA. No standardized validated PA questionnaire or objective activity-monitoring method was used. Therefore, detailed information regarding PA intensity, frequency, activity type, sedentary behavior, occupational activity, and recall period was not systematically available. Because PA represented the primary exposure variable, potential recall bias, social desirability bias, and exposure misclassification should be considered when interpreting the observed associations. Participants were categorized according to World Health Organization recommendations as low PA (<150 min/week), moderate PA (150–300 min/week), and high PA (>300 min/week) [17].

### 2.5. Functional Performance Assessment

Functional performance was evaluated in a subgroup of patients with available Timed Up and Go (TUG) test data (*n* = 214). The TUG test was timed using a Garmin digital stopwatch (Garmin International, Inc., Olathe, KS, USA). TUG testing was not performed systematically in the entire cohort. Rather, it was conducted in patients for whom functional assessment was clinically indicated and recorded as part of routine primary care. Therefore, the TUG subgroup may differ systematically from the full study population, creating potential selection bias and limiting generalizability of the functional-performance findings. Baseline characteristics of the TUG subgroup were compared with those of the overall cohort to assess potential subgroup differences.

### 2.6. Outcome Measures

The primary outcome was LDL-C concentration (mmol/L), used as a routine marker of cardiovascular risk. LDL-C values were obtained from routine laboratory measurements performed in certified clinical laboratories. The secondary outcome was functional performance assessed using the Timed Up and Go (TUG) test. This outcome was analyzed in the subgroup of patients with available TUG data (*n* = 214).

### 2.7. Statistical Analysis

Statistical analyses were conducted using SPSS IBM SPSS Statistics for Windows, version 29 (IBM Corp., Armonk, NY, USA). Continuous variables are presented as the mean ± standard deviation (SD), and categorical variables as frequencies and percentages. Normality of continuous variables and model residuals was assessed using visual inspection of histograms and Q–Q plots together with the Shapiro–Wilk test. Homogeneity of variance across PA groups was assessed using Levene’s test. Baseline characteristics were compared across PA categories using one-way analysis of variance (ANOVA) for normally distributed continuous variables and Kruskal–Wallis tests for non-normally distributed continuous variables. Categorical variables were compared using chi-square tests. Differences in LDL-C concentrations across PA groups were assessed using one-way ANOVA followed by Tukey post hoc tests for pairwise comparisons when assumptions were met. If assumptions for parametric testing were not met, the Kruskal–Wallis test was used followed by Dunn’s post hoc test with Bonferroni correction for pairwise comparisons. Multivariable linear regression analysis was conducted to assess associations between PA and LDL-C concentrations after adjustment for age, sex, body mass index (BMI), and diabetes mellitus. PA was entered into the regression model as a categorical variable, with low PA used as the reference category. Moderate PA and high PA were included as indicator variables to estimate their associations with LDL-C relative to the low PA group. Covariates were selected a priori based on clinical relevance, biological plausibility, availability in the routine primary care dataset, and their established associations with lipid metabolism and cardiovascular risk. A univariate screening threshold of *p* < 0.2 was not used because the aim was to adjust for clinically relevant covariates rather than to construct a prediction model based on statistical selection. In the subgroup with available TUG data, an exploratory multivariable linear regression analysis was performed to assess associations between PA category and TUG performance after adjustment for age, sex, BMI, diabetes mellitus, and arterial hypertension. PA was again entered as a categorical variable, with low PA used as the reference category. Because TUG assessment was available only in the clinically assessed subgroup, this analysis was considered exploratory. Lipid-lowering medication use, smoking status, socioeconomic status, dietary variables, alcohol use, cardiovascular disease history, thyroid disease, and renal disease were not systematically available and therefore could not be included in the adjusted models. Regression coefficients, 95% confidence intervals (CIs), and *p*-values were reported. Multicollinearity was assessed using variance inflation factors (VIFs), with values below 2 indicating acceptable levels. Missing data were minimal (<5%) and were not expected to affect the robustness of the analysis. Sensitivity analysis was not considered necessary because the proportion of missing data was low (<5%).

## 3. Results

### 3.1. Participant Flow and Baseline Characteristics

**Figure 1:**
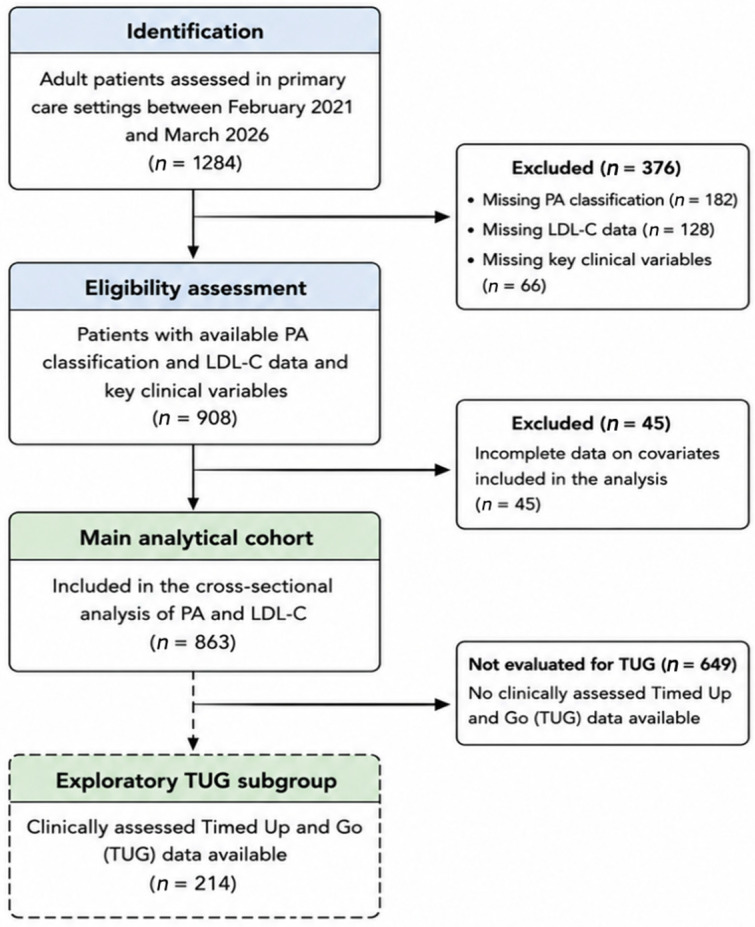
Flowchart of participant selection for the study. The diagram illustrates the identification of adult primary care patients, eligibility assessment, inclusion in the main analytical cohort with available PA and LDL-C data (*n* = 863), and inclusion in the exploratory subgroup with available Timed Up and Go (TUG) functional performance data (*n* = 214).

The participant-selection process is presented in Figure 1. A total of 1284 patient records were initially screened. After exclusion of records with incomplete data or failure to meet inclusion criteria, 863 adult primary care patients were included in the final analysis. The mean age of the overall cohort was 52.4 ± 14.8 years, and 53.3% of participants were female. Baseline demographic and clinical characteristics according to PA category are presented in Table 1. Lower PA categories were associated with older age, higher BMI, higher prevalence of arterial hypertension and diabetes mellitus, and higher LDL-C concentrations across groups. Functional-performance data were available only in the exploratory subgroup with available TUG assessment.

### 3.2. Characteristics of the TUG Subgroup

Functional-performance data assessed using the Timed Up and Go (TUG) test were available in the exploratory subgroup of 214 patients. Because TUG testing was not systematically performed in the entire cohort and was conducted only when clinically indicated and available in routine primary care, the TUG subgroup may differ systematically from the full study population. Baseline characteristics of the full cohort and the TUG subgroup are presented in Table 2. The TUG subgroup demonstrated some clinically relevant differences compared with the overall cohort, including older age, higher BMI, and higher prevalence of arterial hypertension and diabetes mellitus. Therefore, findings related to functional performance should be interpreted cautiously and should not be generalized to the full cohort without qualification.

### 3.3. LDL-C Across Physical Activity Levels

LDL-C concentrations across PA categories are presented in Table 3. Higher PA categories were associated with lower LDL-C concentrations across groups (*p* < 0.001). Mean LDL-C concentrations were 3.68 ± 1.05 mmol/L in the low PA group, 3.39 ± 0.97 mmol/L in the moderate PA group, and 3.12 ± 0.89 mmol/L in the high PA group. Post hoc pairwise comparisons demonstrated significantly lower LDL-C concentrations in both the moderate and high PA groups compared with the low PA group. LDL-C concentrations were also significantly lower in the high PA group compared with the moderate PA group. An overall group comparison demonstrated significant differences across PA categories (F(2860) = 18.72; *p* < 0.001). The observed effect size (η^2^ = 0.041) indicated a small-to-moderate association between PA category and LDL-C concentration.

### 3.4. Functional Performance

Functional-performance findings in the exploratory TUG subgroup are presented in Table 4. Higher PA categories were associated with lower TUG times across groups (*p* < 0.001). Mean TUG times were 11.8 ± 2.9 s in the low PA group, 9.7 ± 2.1 s in the moderate PA group, and 8.4 ± 1.8 s in the high PA group. Post hoc comparisons demonstrated significantly lower TUG times in the moderate and high PA groups compared with the low PA group. TUG times were also significantly lower in the high PA group compared with the moderate PA group. Because TUG assessment was available only in the clinically assessed exploratory subgroup, these findings should be interpreted cautiously and should not be generalized to the full study population without qualification.

### 3.5. Multivariable Regression Analysis

Results of the multivariable regression analysis are presented in Table 5. After adjustment for age, sex, body mass index (BMI), and diabetes mellitus, higher PA categories remained significantly associated with lower LDL-C concentrations. Compared with the low PA group, both moderate and high PA categories demonstrated significant associations with lower LDL-C concentrations after multivariable adjustment. The strongest association was observed in the high PA group. Regression coefficients, 95% confidence intervals, standardized β coefficients, and *p*-values are presented in Table 5. Although clinically relevant covariates were included in the adjusted model, residual confounding from unavailable variables cannot be excluded.

### 3.6. Exploratory Regression Analysis of Functional Performance

Results of the exploratory multivariable regression analysis of functional performance are presented in Table 6. After adjustment for age, sex, body mass index (BMI), diabetes mellitus, and arterial hypertension, higher PA categories remained significantly associated with lower TUG times in the exploratory subgroup with available TUG assessment. Compared with the low PA group, both moderate and high PA categories demonstrated significant associations with lower TUG times after multivariable adjustment. The magnitude of the association was greater in the high PA group. Regression coefficients, 95% confidence intervals, standardized β coefficients, and *p*-values are presented in Table 6. Because TUG assessment was available only in the clinically assessed exploratory subgroup, these findings should be interpreted cautiously and should not be generalized to the full study population without qualification.

## 4. Discussion

### 4.1. Principal Findings

In this cross-sectional observational study of a real-world primary care population, higher self-reported PA categories were associated with lower LDL-C concentrations across groups. In the exploratory subgroup with available TUG assessment, higher PA categories were also associated with lower TUG times, suggesting better functional performance. These findings remained significant after multivariable adjustment for clinically relevant covariates. However, because of the observational cross-sectional design and the potential for residual confounding, causality and directionality cannot be established. In addition, the functional-performance findings should be interpreted cautiously because TUG assessment was available only in the clinically assessed exploratory subgroup and was not systematically performed in the entire cohort. The association between PA and LDL-C remained significant after adjustment for age, sex, BMI, and diabetes mellitus. However, this finding should be interpreted cautiously because adjustment was limited to variables systematically available in the routine primary care dataset. Several important potential confounders, including lipid-lowering medication use, smoking status, socioeconomic status, dietary factors, alcohol use, cardiovascular disease history, thyroid disease, and renal disease, were not systematically available. These factors may influence LDL-C levels, PA behavior, and overall cardiovascular risk. Therefore, the observed LDL-C differences, including the approximately 0.56 mmol/L difference between the low and high PA groups, should be interpreted as cross-sectional associations rather than evidence that PA independently caused lower LDL-C.

### 4.2. Comparison with Existing Literature

The present findings are consistent with previous evidence showing that higher PA levels are associated with more favorable lipid profiles and lower cardiovascular risk [1,2,3]. Similarly, contemporary observational studies have reported associations between PA, metabolic health, insulin sensitivity, and lipid metabolism [6,7]. In addition to metabolic outcomes, previous studies have consistently shown that higher PA levels are associated with better mobility, balance, and functional performance, particularly in aging populations [12,13]. The lower TUG times observed across higher PA categories in the present study are broadly consistent with earlier reports linking PA with better physical function and lower risk of functional decline [18]. Importantly, the present findings should be interpreted within the context of the observational cross-sectional design. Although the observed associations are consistent with the prior literature, causality and directionality cannot be established from the present analysis.

### 4.3. Physiological Mechanisms

The observed associations between PA and LDL-C concentrations in the present study are biologically plausible and are broadly consistent with physiological pathways previously discussed in the literature, including lipoprotein metabolism, insulin sensitivity, vascular function, autonomic regulation, and systemic inflammatory processes [9,10,11,19]. Similarly, previous studies have suggested that regular PA may be associated with functional capacity through mechanisms related to mobility, balance, muscle function, and cardiovascular adaptation. However, the present study did not directly assess physiological or mechanistic variables. Therefore, no conclusions regarding underlying mechanisms can be drawn from the current data.

### 4.4. Real-World Context and Behavioral Gap

Despite extensive evidence supporting the health relevance of PA, long-term adherence to recommended PA levels remains inconsistent in real-world primary care populations [4,5,14,20]. A substantial gap persists between recommended and actual PA behavior, particularly among individuals with increased cardiometabolic risk. These real-world challenges suggest that sustained PA behavior may be influenced by multiple behavioral, clinical, environmental, and lifestyle-related factors beyond awareness of health recommendations alone. From a clinical perspective, this highlights the importance of considering long-term behavioral sustainability when interpreting PA-related associations in routine primary care settings.

### 4.5. Hypothesis-Generating Regulatory Interpretation

The observed associations may also be considered from a cautious hypothesis-generating perspective. Sustained physical activity (PA) behavior may reflect broader self-regulatory capacity, including the ability to maintain adaptive health-related behaviors over time. This interpretation is broadly consistent with contemporary perspectives on interoception, allostasis, autonomic regulation, and stress-related regulatory load [21,22,23].

However, these regulatory processes were not directly measured in the present study. Therefore, this interpretation should be considered theoretical and hypothesis-generating rather than an empirical explanation of the observed findings.

Future studies incorporating objective PA monitoring together with a direct assessment of regulatory processes, including physiological stress markers, autonomic function (e.g., heart rate variability), recovery dynamics, perceived stress, and behavioral adherence patterns, are needed to evaluate these hypotheses.

### 4.6. Clinical and Public Health Implications

The present findings support the clinical relevance of PA assessment in routine primary care. Higher self-reported PA categories were associated with lower LDL-C concentrations in the full cohort and with lower TUG times in the exploratory subgroup with available functional assessment. From a clinical perspective, routine assessment of PA status may provide useful contextual information regarding cardiometabolic risk profile and functional status in primary care populations. However, because of the observational cross-sectional design and the potential for residual confounding, the present findings should not be interpreted as evidence that increasing PA alone would necessarily reduce LDL-C concentrations or improve functional performance. In addition, the functional-performance findings should be interpreted cautiously because TUG assessment was available only in the clinically assessed exploratory subgroup. Longitudinal and interventional studies are needed to determine whether changes in PA are associated with subsequent changes in lipid profile and functional outcomes under real-world primary care conditions.

### 4.7. Limitations

Several limitations should be acknowledged. First, the cross-sectional observational design precludes conclusions regarding causality or directionality. Second, PA was assessed using patient self-reports during a routine clinical evaluation. No standardized validated PA questionnaire or objective activity-monitoring method was used. Therefore, recall bias, social desirability bias, exposure misclassification, and overestimation of PA levels are possible. In addition, detailed information regarding PA intensity, frequency, activity type, sedentary behavior, occupational activity, and recall period was not systematically available. Third, although the regression analyses adjusted for clinically relevant covariates, including age, sex, BMI, diabetes mellitus, and arterial hypertension, in the TUG analysis, several potentially important confounding variables were not systematically available. These included lipid-lowering medication use, smoking status, socioeconomic status, dietary variables, alcohol use, cardiovascular disease history, thyroid disease, and renal disease. The absence of lipid-lowering medication data is particularly relevant for interpretation of LDL-C concentrations because treatment status may substantially influence measured LDL-C values independently of PA behavior. Therefore, residual confounding cannot be excluded. Fourth, TUG assessment was available only in a clinically assessed exploratory subgroup and was not systematically performed in the entire cohort. Therefore, subgroup-related selection bias and limited generalizability of the functional-performance findings should be considered when interpreting these results. Finally, regulatory processes were not directly measured or tested in the present study. Therefore, the regulatory interpretation discussed in this manuscript should be viewed only as a theoretical and hypothesis-generating perspective for future research rather than as an evidence-based mechanistic explanation. Despite these limitations, this study also has important strengths. The analysis was based on a relatively large real-world primary care cohort and reflects routine clinical conditions, supporting external validity. In addition, the combined assessment of cardiometabolic and functional outcomes provides a broader perspective on health status in primary care populations.

### 4.8. Future Directions

Future studies should incorporate standardized validated PA questionnaires together with objective PA assessment methods, such as accelerometry or wearable-device monitoring [24]. A more detailed characterization of PA intensity, frequency, activity type, sedentary behavior, occupational activity, and long-term behavioral adherence would further strengthen future analyses. Longitudinal and interventional study designs will be important for evaluating temporal relationships between PA, LDL-C concentrations, functional performance, and cardiometabolic risk profile [25]. Future research should also include a direct assessment of regulatory processes, including heart rate variability, physiological stress markers, recovery dynamics, perceived stress, behavioral adherence patterns, and objective PA monitoring, before conclusions regarding regulatory efficiency can be considered. Because TUG performance may be associated with later functional decline in older adults, future longitudinal studies should evaluate whether PA-related differences in TUG performance are associated with clinically meaningful long-term outcomes [26].

## 5. Conclusions

In this real-world primary care population, higher self-reported PA categories were associated with lower LDL-C concentrations. Higher PA categories were also associated with lower TUG times in the exploratory subgroup with available functional assessment. Because of the cross-sectional observational design, these findings do not establish causality or directionality. The LDL-C findings should be interpreted cautiously because residual confounding from unavailable variables, including lipid-lowering medication use, smoking status, diet, socioeconomic status, and other cardiovascular risk factors, cannot be excluded. The functional-performance findings should be interpreted cautiously because TUG assessment was not performed systematically in the full cohort. The present findings support the clinical relevance of routine PA assessment in primary care while highlighting the need for future longitudinal and interventional studies using objective PA measures, a more complete confounder assessment, and direct measures of regulatory processes. Regulatory efficiency may represent a hypothesis-generating construct for future research on sustained PA behavior, but it was not directly measured or tested in this study and should not be interpreted as a conclusion of the present analysis.

## Figures and Tables

**Table 1:** Baseline demographic and clinical characteristics according to physical activity categories. Baseline demographic and clinical characteristics of the study population stratified according to physical activity (PA) categories.

Variable	Low PA (*n* = 249)	Moderate PA (*n* = 353)	High PA (*n* = 261)	*p*-Value
Age (years), mean ± SD	64.8 ± 11.2	58.1 ± 10.4	52.6 ± 9.7	<0.001
Female sex, *n* (%)	133 (53.4)	182 (51.6)	145 (55.6)	0.81
BMI (kg/m^2^), mean ± SD	31.4 ± 5.2	28.7 ± 4.6	26.1 ± 3.9	<0.001
LDL-C (mmol/L), mean ± SD	3.68 ± 1.05	3.39 ± 0.97	3.12 ± 0.89	<0.001
Arterial hypertension, *n* (%)	139 (55.8)	144 (40.8)	75 (28.7)	<0.001
Diabetes mellitus, *n* (%)	59 (23.7)	52 (14.7)	26 (10.0)	<0.001
Timed Up and Go (TUG), mean ± SD *	11.8 ± 2.9	9.7 ± 2.1	8.4 ± 1.8	<0.001

Continuous variables were compared using one-way ANOVA or the Kruskal–Wallis test where appropriate. Categorical variables were compared using the chi-square (χ^2^) test. Abbreviations: BMI, body mass index; LDL-C, low-density lipoprotein cholesterol; PA, physical activity; SD, standard deviation; TUG, Timed Up and Go. * Timed Up and Go (TUG) performance data were available only in the clinically assessed subgroup (*n* = 214).

**Table 2:** Comparison of the full cohort and TUG subgroup. Baseline demographic and clinical characteristics comparing the full study cohort with the subgroup of patients with available Timed Up and Go (TUG) assessment.

Variable	Full Cohort (*n* = 863)	TUG Subgroup (*n* = 214)	*p*-Value
Age (years), mean ± SD	52.4 ± 14.8	67.2 ± 10.9	<0.001
Female sex, *n* (%)	460 (53.3)	121 (56.5)	0.41
BMI (kg/m^2^), mean ± SD	28.7 ± 4.9	30.2 ± 5.1	0.002
LDL-C (mmol/L), mean ± SD	3.42 ± 1.01	3.51 ± 0.96	0.18
Arterial hypertension, *n* (%)	358 (41.5)	142 (66.4)	<0.001
Diabetes mellitus, *n* (%)	126 (14.6)	58 (27.1)	<0.001
Low PA, *n* (%)	249 (28.9)	74 (34.6)	0.08
Moderate PA, *n* (%)	353 (40.9)	82 (38.3)	0.52
High PA, *n* (%)	261 (30.2)	58 (27.1)	0.39

Continuous variables were compared using Student’s *t*-test or Mann–Whitney U test as appropriate. Categorical variables were compared using chi-square (χ^2^) tests. Abbreviations: BMI, body mass index; LDL-C, low-density lipoprotein cholesterol; PA, physical activity; SD, standard deviation; TUG, Timed Up and Go. The TUG subgroup consisted of patients in whom functional assessment was clinically indicated and available during routine primary care evaluation.

**Table 3:** LDL-C levels across physical activity categories with post hoc comparisons. Comparison of low-density lipoprotein cholesterol (LDL-C) concentrations across physical activity (PA) categories.

Physical Activity Category	*n*	LDL-C (mmol/L), Mean ± SD	Post Hoc Comparison	Adjusted *p*-Value
Low PA	249	3.68 ± 1.05	Reference	—
Moderate PA	353	3.39 ± 0.97	Moderate vs. Low	<0.001
High PA	261	3.12 ± 0.89	High vs. Low	<0.001
High PA	261	3.12 ± 0.89	High vs. Moderate	0.002

Overall group comparison: F(2860) = 18.72; *p* < 0.001; η^2^ = 0.041. Post hoc comparisons were performed using Bonferroni correction. Abbreviations: LDL-C, low-density lipoprotein cholesterol; PA, physical activity; SD, standard deviation.

**Table 4:** Timed Up and Go performance across physical activity categories in the TUG subgroup. Comparison of functional performance assessed by the Timed Up and Go (TUG) test across physical activity (PA) categories within the clinically assessed subgroup.

Physical Activity Category	*n*	TUG (s), Mean ± SD	Post Hoc Comparison	Adjusted *p*-Value
Low PA	74	11.8 ± 2.9	Reference	—
Moderate PA	82	9.7 ± 2.1	Moderate vs. Low	<0.001
High PA	58	8.4 ± 1.8	High vs. Low	<0.001
High PA	58	8.4 ± 1.8	High vs. Moderate	0.004

Overall group comparison: F(2211) = 26.45; *p* < 0.001. Post hoc comparisons were performed using Bonferroni correction. Values are presented as the mean ± standard deviation. Abbreviations: PA, physical activity; TUG, Timed Up and Go; SD, standard deviation. TUG assessment was available only in a clinically assessed subgroup and should therefore be interpreted as an exploratory subgroup analysis.

**Table 5:** Multivariable linear regression analysis for LDL-C. Multivariable linear regression analysis evaluating independent associations between physical activity (PA) categories and low-density lipoprotein cholesterol (LDL-C).

Variable	β Coefficient	95% CI	Standardized β	*p*-Value
Moderate PA vs. low PA	−0.29	−0.39 to −0.18	−0.18	<0.001
High PA vs. low PA	−0.54	−0.66 to −0.42	−0.31	<0.001
Age (years)	0.013	0.009 to 0.017	0.22	<0.001
Male sex	0.07	−0.03 to 0.17	0.05	0.18
BMI (kg/m^2^)	0.029	0.019 to 0.039	0.24	<0.001
Diabetes mellitus	0.17	0.05 to 0.29	0.11	0.006

Overall regression model: R^2^ = 0.18; *p* < 0.001. Low PA category was used as the reference category. Abbreviations: BMI, body mass index; CI, confidence interval; LDL-C, low-density lipoprotein cholesterol; PA, physical activity. The regression model was adjusted for age, sex, BMI, and diabetes mellitus. Male sex was coded relative to female sex as the reference category.

**Table 6:** Exploratory multivariable linear regression analysis for TUG performance. Exploratory multivariable linear regression analysis evaluating associations between physical activity (PA) categories and Timed Up and Go (TUG) performance within the clinically assessed subgroup.

Variable	β Coefficient	95% CI	Standardized β	*p*-Value
Moderate PA vs. low PA	−1.24	−1.89 to −0.59	−0.26	<0.001
High PA vs. low PA	−2.11	−2.83 to −1.39	−0.39	<0.001
Age (years)	0.07	0.04 to 0.10	0.31	<0.001
Male sex	−0.21	−0.62 to 0.20	−0.06	0.31
BMI (kg/m^2^)	0.11	0.06 to 0.16	0.27	<0.001
Diabetes mellitus	0.68	0.24 to 1.12	0.15	0.003
Arterial hypertension	0.53	0.11 to 0.95	0.12	0.014

Overall regression model: R^2^ = 0.29; *p* < 0.001. Low PA category was used as the reference category. Abbreviations: BMI, body mass index; CI, confidence interval; PA, physical activity; TUG, Timed Up and Go. This analysis was exploratory because TUG assessment was available only in the clinically assessed subgroup (*n* = 214). The regression model was adjusted for age, sex, BMI, diabetes mellitus, and arterial hypertension.

## Data Availability

The datasets used and/or analyzed during the current study are available from the corresponding author on reasonable request.

## Supplementary Materials

The following supporting information can be downloaded at: https://www.mdpi.com/article/10.3390/healthcare14111522/s1, Table S1: STROBE Checklist for Reporting of Observational Studies.

## Abbreviations

PA, physical activity
LDL-C, low-density lipoprotein cholesterol
TUG, Time up and Go
