# Supplementary material for: Real-World Associations Between Physical Activity, LDL Cholesterol, and Functional Performance in Primary Care: A Cross-Sectional Study
Source: Healthcare (Basel). 2026 May 30;14(11):1522. doi: 10.3390/healthcare14111522 (PMC13257027; doi:10.3390/healthcare14111522)
Supplement: Supplementary file 1 [file healthcare-14-01522-s001.zip › healthcare-4286824-supplementary.pdf]

healthcare

MDPI

Supplementary Table S1. STROBE Checklist for Reporting of Observational Studies.

**Section/Item**	**Recommendation**	**Reported on Page(s)**
Title and Abstract	Indicate the study design in the title or abstract	Title page and Abstract
Background/Rationale	Explain the scientific background and rationale	Introduction
Objectives	State specific objectives	Introduction
Study Design	Present key elements of study design early in the paper	Methods 2.1
Setting	Describe the setting, locations, and relevant dates	Methods 2.1–2.3
Participants	Give eligibility criteria and selection methods	Methods 2.2
Variables	Clearly define outcomes, exposures, predictors, confounders	Methods 2.3–2.6
Data Sources/Measurement	Describe data sources and assessment methods	Methods 2.3–2.5
Bias	Describe efforts to address potential sources of bias	Methods 2.3, 2.7; Discussion
Study Size	Explain how the study size was determined	Methods 2.2
Quantitative Variables	Explain handling of quantitative variables	Methods 2.7
Statistical Methods	Describe statistical methods and confounder adjustment	Methods 2.7

Section/Item	Recommendation	Reported on Page(s)
Participants	Report numbers of individuals at each study stage	Results 3.1; Figure 1
Descriptive Data	Present participant characteristics	Table 1; Table 2
Outcome Data	Report outcome measures	Results 3.3–3.6; Tables 3–6
Main Results	Provide unadjusted and adjusted estimates	Tables 5–6
Other Analyses	Report subgroup or exploratory analyses	Results 3.4 and 3.6
Key Results	Summarize key findings	Discussion
Limitations	Discuss limitations and potential bias	Discussion
Interpretation	Provide cautious interpretation of findings	Discussion and Conclusions
Generalizability	Discuss external validity/generalizability	Discussion
Funding	Describe funding sources	Funding Statement

STROBE = Strengthening the Reporting of Observational Studies in Epidemiology.
